# Hospitalist System under the Covid-19 Pandemic: The Perspective of Value Co-creation: Yu Hua Yan

**DOI:** 10.1093/eurpub/ckac131.289

**Published:** 2022-10-25

**Authors:** Y Yan

**Affiliations:** Superintendent Office, Tainan Municipal Hospital, Tainan, Taiwan

## Abstract

**Background:**

The core spirit of the Hospitalist system aims to set up dedicated wards, integrate physician manpower, focus on whole-person care, in order to cope with the aging population and Covid-19 pandemic, and to ensure that both parties, the medical personnel and patients, can provide or receive complete medical care. As the Taiwan medical system is facing a paradigm shift, the Taiwan hospitalist system will play an essential role in the transition as moving forward to provide professional care for inpatients.

**Methods:**

Hospitalists from 12 hospitals across Taiwan completed a cross-sectional survey. The target population was identified through Taiwan Doctors and Nurses. Survey questionnaire was accessed by 342, incomplete response (18) were excluded and 324 completed responses were analysed.

**Results:**

That the higher the cognition of medical staff on whole-person care, the higher the motivation to participate in cross-team cooperation (F = 35.914, p < 0.001); when the motivation to participate in cross-team cooperation was higher, the behavior of participating in whole-person care also increased. Will be higher (F = 36.483, p < 0.001); whole-person care behavior participation behavior has a significant impact on value creation (F = 21.068, p < 0.001)

**Conclusions:**

As the Taiwan medical system is facing a paradigm shift, the Taiwan hospitalist system will play an essential role in the transition as moving forward to provide professional care for inpatients. This change will make possible the improvement of patient safety and quality medical care. The research results can be provided for reference in European and American countries.

**Key messages:**

• A hospitalist support system is essential for establishing an efficient medical environment and reducing administrative work, which can help hospitals introduce a hospitalist system.

• To build a more stable and sustainable system, it is necessary to create a systemic operational foundation for proceeding with this new hospitalist system.

